# The gut microbiota: emerging biomarkers and potential treatments for infertility-related diseases

**DOI:** 10.3389/fcimb.2024.1450310

**Published:** 2024-09-26

**Authors:** Min Wang, Lian-Wen Zheng, Shuai Ma, Dong-Hai Zhao, Ying Xu

**Affiliations:** ^1^ Department of Obstetrics and Gynecology, The Second Hospital of Jilin University, Changchun, China; ^2^ Department of Pathology, Jilin Medical University, Jilin, China

**Keywords:** gut microbiota, infertility, polycystic ovary syndrome, endometriosis, premature ovarian failure, biomarkers, therapeutics

## Abstract

Infertility is a disease of impaired fertility. With socioeconomic development, changes in human lifestyles, and increased environmental pollution, the problem of low human fertility has become increasingly prominent. The incidence of global infertility is increasing every year. Many factors lead to infertility, and common female factors include tubal factors, ovulation disorders, endometriosis, and immune factors. The gut microbiota is involved in many physiological processes, such as nutrient absorption, intestinal mucosal growth, glycolipid metabolism, and immune system regulation. An altered gut flora is associated with female infertility disorders such as polycystic ovary syndrome (PCOS), endometriosis (EMs), and premature ovarian failure (POF). Dysbiosis of the gut microbiota directly or indirectly contributes to the development of female infertility disorders, which also affect the homeostasis of the gut microbiota. Identifying the etiology and pathogenesis of infertility in patients is the focus of reproductive medicine physicians. We studied the developmental mechanism between the gut microbiota and PCOS, EMs, and POF from a new perspective, providing new ideas for diagnosing and treating female infertility diseases and specific reference values for eugenics.

## 1 Introduction

The aging process of female ovaries is accelerating, and the incidence of infertility is significantly increasing at a younger age (Inhorn and Patrizio, 2015). Delayed childbearing has become a global problem. The WHO predicts that infertility will become the third most important disease of the 21st century after tumors and cardiovascular disease (Carson and Kallen, 2021). Clinical conditions that lead to female infertility include PCOS, EMs, and POF (American College of Obstetricians and Gynecologists Committee on Gynecologic Practice and Practice Committee, 2014; Gu et al., 2022; Salmeri et al., 2024). Reduced human fertility is not simply a reproductive health issue but also raises a variety of social, economic, and family issues. Approximately 100 trillion microorganisms colonize the human gastrointestinal tract, and gut microorganisms form interdependent symbioses with their hosts, affecting normal physiology and susceptibility to disease (Illiano et al., 2020). James et al. proposed that the gut microbiota plays a vital role in the pathogenesis of various estrogen-dependent diseases and proposed the concept of the “estrogen-gut microbiota axis.” The gut microbiota regulates estrogen by secreting β-glucuronidase (Flores et al., 2012). Disruption of this process by dysbiosis of the gut microbiota results in a decrease in circulating estrogen. Alterations in circulating estrogen can lead to the development of diseases such as obesity, metabolic syndrome, PCOS, EMs, and decreased fertility (Saunders and Horne, 2021). With the increasing development and improvement of microbiome research, the involvement of the gut microbiota in the curative mechanism of female infertility diseases such as PCOS, EMs, and POF deserves further investigation, which is highly important in guiding the improvement of female infertility and fertility (Dinsdale and Crespi, 2021).

## 2 Gut microbiota

### 2.1 Composition of the gut microbiota

The gut microbiota is a general term for all microorganisms colonizing the human gastrointestinal tract, with a wide variety of species, large numbers, and complex functions, known as the “second genome of the human body” (Adak and Khan, 2019). The proportion of Firmicutes and Bacteroidetes in the gut microbiota in the human body is as high as 90%, followed by Actinomycetes, Proteobacteria, and Fusobacteria, which are involved in the maintenance of the microecological balance in the human body (Zeng et al., 2019). The relationships between the gut microbiota and the host are divided into three major categories: beneficial bacteria, such as Lactobacillus and Bifidobacterium, which help the body digest and absorb toxins, reduce the release of toxins, improve the body’s immune system, alleviate inflammatory reactions, and decrease the incidence of tumors. Harmful bacteria, such as Salmonella and Staphylococcus, increase the toxin content, disrupt the internal environment of the intestine, and increase the incidence of cancer. Harmful bacteria, such as Salmonella and Staphylococcus, can increase the level of toxins and damage the internal environment of the intestine, leading to intestinal diseases and increasing the incidence of cancer; intermediate bacteria, such as Bacteroides and *Escherichia coli* (Liu et al., 2017; McQuade et al., 2019).

### 2.2 Functions of the gut microbiota

**Figure 1:**
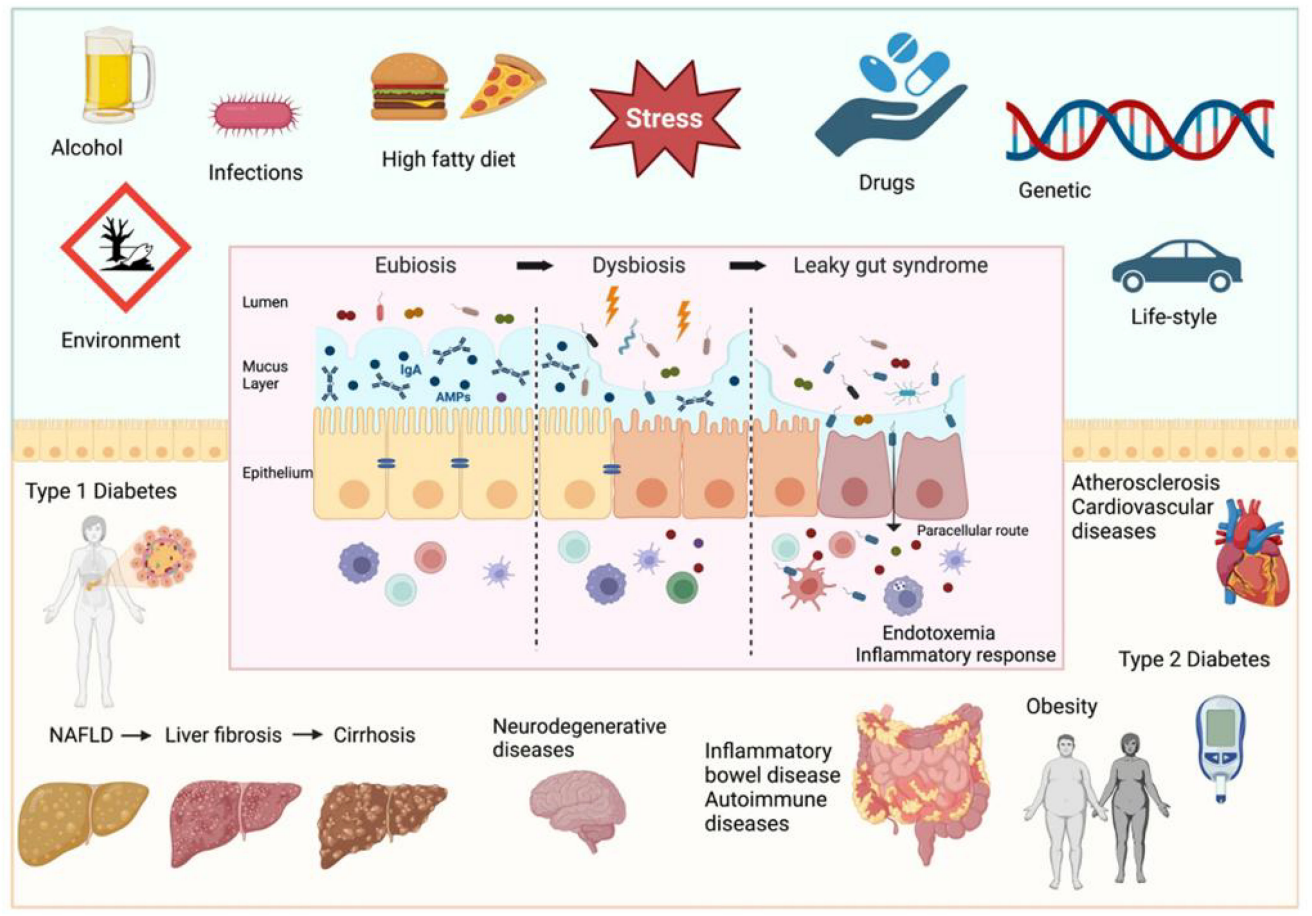
Factors determining intestinal barrier impairment and consequent systemic diseases (Di Vincenzo et al., 2024).

The species and number of microorganisms colonizing the gut vary within a certain range and are in dynamic equilibrium (Zemel, 2017). The gut microbiota plays important roles in human growth and development, metabolism, immunity, and other pathophysiological processes, including the promotion of host immune system maturation, the inhibition of pathogen overgrowth, the regulation of intestinal endocrine function, neural signaling, and the synthesis of neurotransmitters (He and Li, 2020). The gut microbiota not only exerts various effects on the intestinal environment but also regulates distal tissues and organs and is considered to be a mature endocrine organ (Martin-Gallausiaux et al., 2021). With the development of gene sequencing technology, in-depth knowledge of the gut microbiota has increased, and many studies have confirmed that the gut microbiota composition and diversity are altered when dysbiosis occurs and that dysbiosis of the gut microbiota can promote the occurrence and development of diseases through various pathways, such as neuroendocrine and metabolic immunity pathways, in the human body (Di Vincenzo et al., 2024) (**Figure 1**). The gut microbiota has become one of the hotspots of research in medicine, microbiology, genetics, etc (Yan and Charles, 2018). The gut microbiota plays a vital role in female reproductive health. It can be involved in the occurrence of diseases of the female reproductive system by directly or indirectly participating in the regulation of sex hormones, stimulating the production of inflammatory factors, and influencing immune function and metabolic homeostasis (Escobar-Morreale, 2018).

## 3 The gut microbiota and female infertility

Infertility has been a significant challenge in reproductive medicine (Wild, 2002). The leading causes of female infertility include PCOS, EMs, and POF (Torres et al., 2018a). PCOS is one of the most common reproductive endocrine disorders in women of reproductive age, and it is one of the most important causes of ovulatory dysfunction and infertility in women of reproductive age (Shi et al., 2021). The etiology and pathogenesis of PCOS have not been elucidated. PCOS is characterized clinically by irregular menstruation, hyperandrogenism, or hyperandrogenism. Polycystic changes characterize ultrasound-generated ovaries. PCOS is characterized by irregular menstruation, hyperandrogenism or hyperandrogenaemia, and polycystic ovarian changes on ultrasound, with infertility as the primary manifestation (Sadeghi et al., 2022). EMs is a common gynecological disease with a trend of increasing incidence yearly. Pain and infertility caused by EMs are severe threats to women’s physical and mental health (Johnson et al., 2017). The natural pregnancy rate of patients with EMs decreases annually with increasing postoperative time, and ART also leads to low implantation rates due to the poor quality of oocytes and embryos in patients with EMs (Bailey and Coe, 2002; Flores et al., 2012). POF is a reproductive endocrine disease with a complex etiology, with genetic, immunologic, environmental, oxidative stress, chronic inflammation, and other influences that may contribute to the development of POF (Sullivan et al., 2016). The specific etiology of POF has not been fully elucidated. POF not only affects the reproductive function of patients but also increases the risk of depression, anxiety, cognitive decline, premature death, osteoporosis, and cardiovascular disease (Arroyo et al., 2019). Infertile couples aspire to seek ART to help them conceive, but the treatment process is complex, lengthy, and expensive, further increasing the psychological and financial burden on infertile patients (Tay et al., 2020).

**Figure 2:**
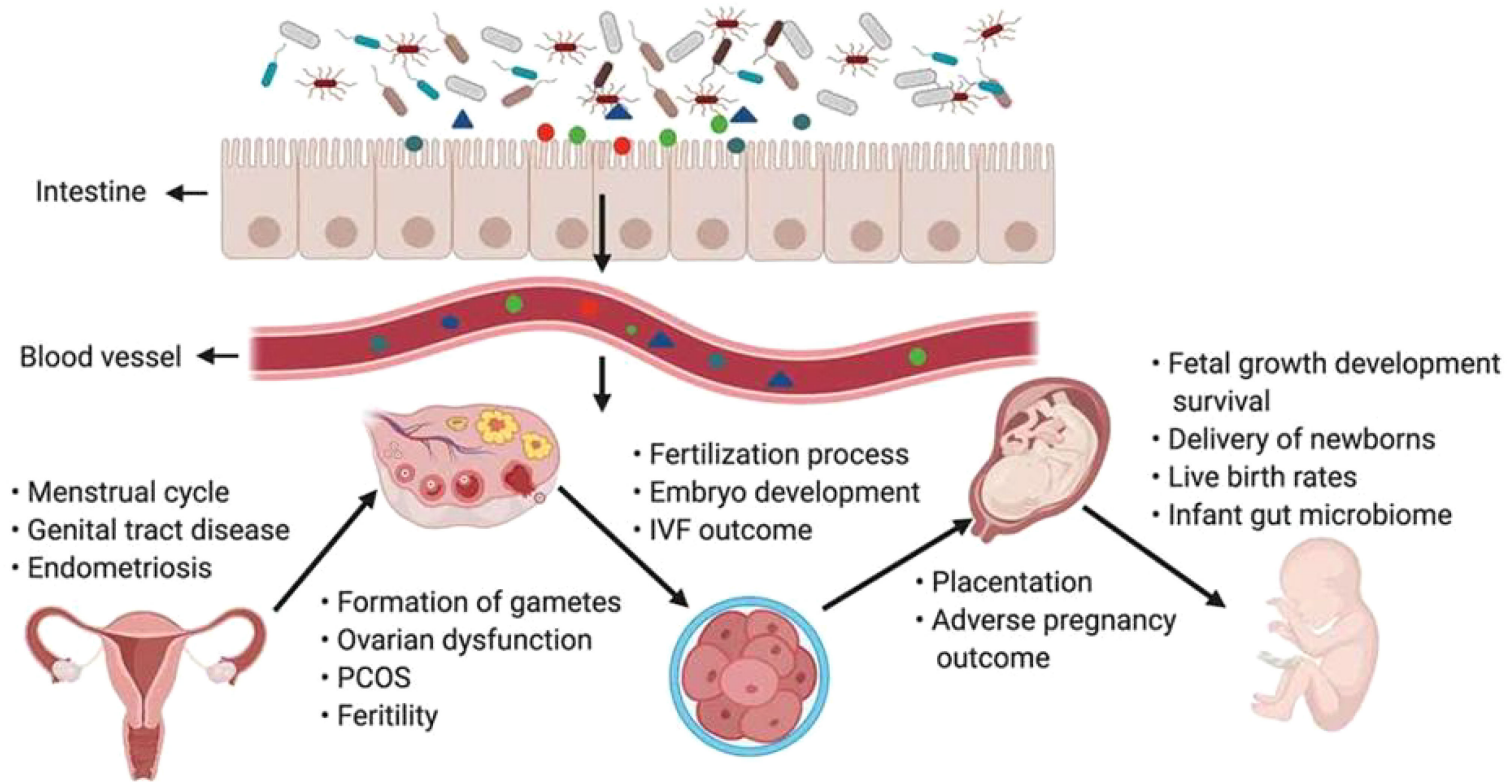
The gut microbiota and its impact on the female reproductive tract, embryo development and pregnancy (Qi et al., 2021).

The body microbiome affects every stage of female reproduction, including follicular and oocyte maturation in the ovary, fertilization, embryo migration, implantation, gestation, and delivery. The gut microbiota is closely associated with the onset and development of reproductive system diseases (Kelley et al., 2016). Disturbances in the gut microbiota increase the production of short-chain fatty acids (SCFAs), lipopolysaccharides (LPS), etc., which influence the secretion of gonadotropins and sex hormones from the central nervous system, including the hypothalamus and pituitary gland, through neural and humoral signaling (Backhed et al., 2004; Grunewald et al., 2019). Many aspects of the female pregnancy process are related to estrogen. The growth and development of the follicle, endometrial hyperplasia, endometrial tolerance, gestational maintenance of the ovarian corpus luteum, and early placental perfusion cannot be achieved without normal estrogen regulation of the body (Baker et al., 2017; Durack and Lynch, 2019). Through a variety of pathways, including the brain−gut signaling axis, endocrine system, metabolic system, and immune system, gut microbiota dysregulation can contribute to the onset and progression of diseases such as PCOS, EMs, and POF and increase the incidence of infertility in women of reproductive age (Qi et al., 2021) (**Figure 2**). Disease can also disrupt the homeostasis of the gut microbiota. Correction of abnormal microbiota may improve reproductive outcomes (Rizk and Thackray, 2021). We focused on the interactions between the gut microbiota and multiple diseases that cause infertility and explored the pathogenesis of the gut microbiota and female infertility to provide ideas for the diagnosis and treatment of female infertility disorders.

## 4 The gut microbiota and PCOS

### 4.1 Overview of PCOS

PCOS is the most common endocrine disorder leading to anovulatory infertility (Zhang et al., 2022). The incidence of PCOS, which has a severe impact on women’s bodies, minds, and families, is increasing annually (Li et al., 2016). PCOS is characterized mainly by metabolic abnormalities and other clinical features, including sparseness or anovulation, hyperandrogenism (HA), polycystic changes in the ovaries, insulin resistance (IR), obesity, chronic low-grade inflammation, and infertility (Masjedi et al., 2019). Patients with PCOS are at increased risk for long-term metabolic disorders such as cardiovascular disease and metabolic syndrome (MS) (Witchel et al., 2022). The Rotterdam criteria are currently the most widely used criteria for the diagnosis of PCOS: (1) clinical manifestations of hyperandrogenism or hyperandrogenaemia; (2) sporadic ovulation or anovulation; (3) polycystic changes of the ovary: ovarian volume ≥10 ml or ultrasound suggests that there are ≥12 follicles with a diameter of 2–9 mm in one or both ovaries; and (4) two of the three itEMs and exclusion of other hyperandrogenic aetiologies (Rao and Bhide, 2020). The four critical pathophysiological alterations in PCOS are excessive carbohydrate intake, hyperandrogenism, hyperinsulinaemia, and inflammation (Li et al., 2022a). The relationship between changes in the gut microbiota and PCOS has been the focus of numerous studies in recent years (Barroso et al., 2020). Alterations in the gut microbiota profile are prevalent in patients with PCOS, and an intestinal microecological imbalance is also closely related to the occurrence and progression of PCOS (Ostadmohammadi et al., 2019).

### 4.2 Mechanisms of gut microbiota involvement in PCOS

**Figure 3:**
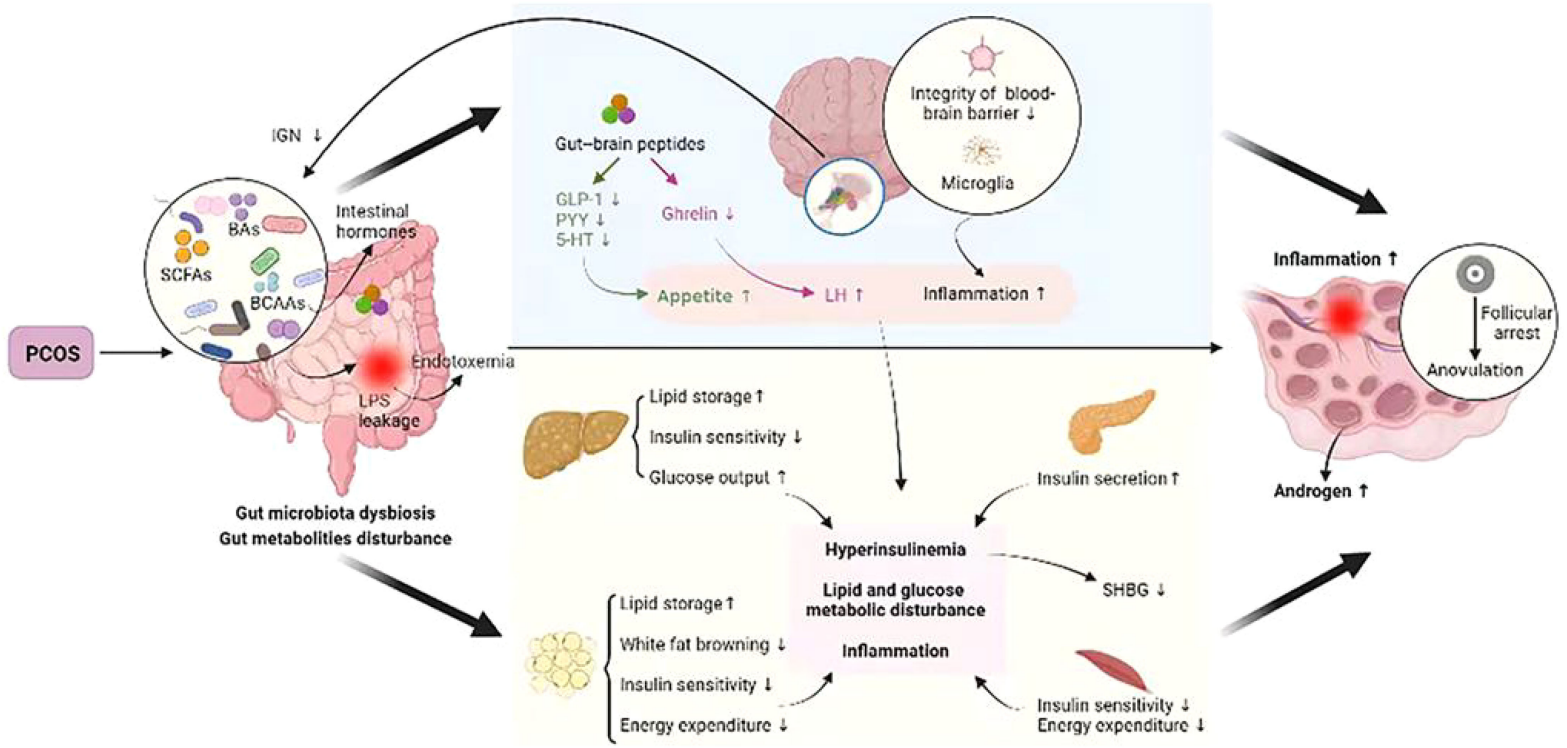
Crosstalk between PCOS and gut metabolites (Zhang et al., 2022).

The gut microbiota is the “endocrine organ” that maintains human health. The microbiota in the gut affects the reproductive endocrine system by interacting with estrogens, androgens, insulin, etc (Lan et al., 2023). The typical features of PCOS include abnormal sex hormone levels, IR, polycystic changes in the ovaries, chronic inflammation, and oxidative stress (Liu et al., 2021; Azizi-Kutenaee et al., 2022). Disturbances in the gut microbiota are involved in endotoxemia, SCFA production, bile acid metabolism, and abnormal ghrelin secretion, and these processes are closely related to the manifestations of HA, IR, chronic inflammatory response, and abnormal ghrelin levels in individuals with PCOS (Chu et al., 2020a). Gut microbiota structural disorders can impair the integrity of the intestinal mucosa, decreasing intestinal barrier function and triggering systemic chronic low-grade inflammation, which in turn leads to endocrine and metabolic disorders that can induce the occurrence and development of PCOS and affect fertility (Tremellen and Pearce, 2012; Insenser et al., 2018). PCOS can lead to gut microbiota dysregulation, and gut microbiota dysregulation exacerbates metabolic and endocrine dysfunction in patients with PCOS. The gut microbiota is involved in the pathogenesis of PCOS by affecting follicular development, sex hormones, and metabolic levels through HA, IR, chronic inflammation, MS, and the gut−brain axis, among other pathologies (Zhang et al., 2022) (**Figure 3**).

#### 4.2.1 Dysregulation of the gut microbiota and HA

HA is the core pathological manifestation of PCOS. Excessive androgen secretion leads to ovarian stromal hyperplasia, peritoneal thickening, and accelerated follicular atresia, which prompts the conversion of testosterone to dihydrotestosterone in peripheral tissues, causing women to exhibit hirsutism and acne and inducing hormonal disorders of the gonadal axis, resulting in abnormalities in follicular growth and development and ovulation (Sun et al., 2019; Torres et al., 2019). Gene sequencing analysis of fecal microorganisms from healthy women, women with PCOM, and patients with PCOS revealed that patients with PCOS had the lowest alpha diversity, followed by patients with PCOM, and that HA, testosteroneemia, and hirsutism were negatively correlated with alpha diversity (Torres et al., 2018b; Jobira et al., 2020). Free testosterone levels are associated with the Firmicutes/Bacteroidetes ratio (Chu et al., 2020b). In PCOS patients, Bacteroidaceae, Raoultella, and Prevotella were positively correlated with testosterone. An elevated abundance of the gut microbiota Bacteroidetes was positively correlated with testosterone, BMI, and inflammatory factors in PCOS patients (Qi et al., 2019). After gut microbiota transplantation from adult male mice into the intestines of immature female mice, the testosterone content in the females was significantly greater than that before transplantation, suggesting that the androgen levels of the mice significantly changed after dysbiosis. Compared with those of normally ovulating women, the gut microbiota of PCOS patients tended to simplify the flora, and the more pronounced this trend was, the greater the androgen levels were in the patients (Zhang et al., 2019a). There is a correlation between HA and the gut microbiota, but the causal relationship between HA and gut microbiota disorders is not clear.

#### 4.2.2 Dysregulation of the gut microbiota and IR

IR plays a vital role in the abnormalities in reproductive function and metabolic disorders caused by PCOS and is closely related to the development of PCOS (Sjogren et al., 2009; Siristatidis et al., 2015). IR levels are associated with the gut microbiota (Qi et al., 2019). The gene count of the gut microbiota was found to be negatively correlated with IR levels (Le Chatelier et al., 2013). Dysregulation of the gut microbiota induces IR, which leads to an imbalance of material and energy metabolism in the body and the activation of chronic inflammatory and immune response systems in the body and affects insulin receptor sensitivity (Zheng et al., 2021). A significant reduction in the number of Prevotella was found in PCOS patients with IR compared with that in PCOS patients without IR, and the number of Prevotella was negatively correlated with elevated clinical parameters such as IR, sex hormones, and inflammation (He and Li, 2021). Dysregulation of the gut microbiota in PCOS patients can lead to increased intestinal mucosal permeability, increased incidence of intestinal villus destruction and enteritis, entry of branched-chain amino acids into the body’s circulation, activation of the body’s immune system and inflammatory mediator system, reduced insulin receptor sensitivity, increased insulin levels, and increased insulin and androgen production through positive feedback; thus, androgens further increase the content and interfere with the regular ovulation of the ovary (Ding et al., 2021). IR levels are correlated with gut microbiota disorders, which can cause the activation of relevant inflammatory signaling pathways in the body, thereby triggering the impairment of the insulin signaling pathway and leading to IR (Saad et al., 2016). Improving the gut microbiota is helpful for improving IR and ovulation function in individuals with PCOS (Helli et al., 2022).

#### 4.2.3 Gut microbiota dysregulation and chronic inflammation

A chronic inflammatory state exists in patients with PCOS (Mohadetheh, 2019). The gut microbiota-mediated inflammatory state is highly important for the pathological process of PCOS (Schmidt, 2015). Wadsworthia is a pathogen associated with the proinflammatory response and is closely related to the development of several inflammatory diseases. As detected by 16S rDNA sequencing, wadsworthia levels were greater in the PCOS fecal transplantation group than in the healthy fecal transplantation group, suggesting that wadsworthia is involved in the pathogenesis of PCOS through the inflammatory process. Patients with PCOS exhibit gut microbiota dysregulation, chronic inflammation, and IR, and the degree of change in the gut microbiota is related to the degree of inflammation and the level of IR (Atarashi et al., 2013; Karlsson et al., 2013). The activity of the gut microbiota was increased in PCOS patients, and the levels of dimethylamine and N-acetylglycoprotein in the blood were significantly greater than those in ordinary women. The elevated levels of dimethylamine and N-acetylglycoprotein demonstrated chronic inflammation in PCOS patients, suggesting that the inflammatory state of PCOS patients is associated with gut microbiota dysregulation (Sun et al., 2012). Patients with PCOS have elevated levels of B. vulgatus in the gut; decreased levels of intestinal bacterial metabolites, bile acids, taurocholate, ursodeoxycholic acid, and glycodeoxycholic acid; and decreased levels of IL-22. The gut microbiota influences the pathologic process of PCOS through the B. vulgatus−bile acid−IL-22 axis. Inflammatory signaling pathways crosstalk with insulin signaling pathways, and endotoxemia due to gut microbiota dysregulation is a crucial contributor to the inflammatory state, IR, and the development of obesity (Cani et al., 2007; Copps and White, 2012). Dysregulation of gut microbiota levels due to chronic inflammation is one of the pathogenetic mechanisms of PCOS (Liang et al., 2020). Increased LPS entry into the bloodstream promotes the expression of inflammatory mediators such as TNF-α, IL-6, and other inflammatory mediators, which leads to IR, which in turn is involved in HA and metabolic abnormalities in PCOS patients and prevents normal follicular development (Lindheim et al., 2017).

#### 4.2.4 Dysregulation of the gut microbiota and MS

The gut microbiota is closely related to human metabolic disorders, and the occurrence and development of various endocrine and metabolic diseases are affected by the structural dynamics of the gut microbiota (Zhou et al., 2020). MS is a pathological state in which the metabolism of carbohydrates, fats, and proteins in humans is out of order, and it is a group of complex metabolic disorders that mainly manifest as hyperglycemia, hyperlipidemia, hypertension, and obesity (Coviello et al., 2006). The incidence of MS in PCOS patients is greater than that in healthy controls (Behboudi-Gandevani et al., 2016; Hallajzadeh et al., 2018). A high-fat diet alters intestinal microecology and permeability, promoting LPS production by gastrointestinal gram-negative bacteria and inhibiting the production of SCFAs. A significant increase in Bacteroides and *Escherichia coli* of the genus gram-negative bacillus in the intestinal tracts of patients with PCOS leads to an increase in LPS, which serves as a trigger factor that further contributes to IR and obesity. Among SCFAs, acetic acid, propionic acid, and butyric acid play essential roles in the body’s metabolism, with effects such as balancing glucose homeostasis; regulating insulin sensitivity; and exerting anti-inflammatory, anticancer, and immunomodulatory effects (Maslowski et al., 2009). Disturbances in the gut microbiota lead to a decrease in circulating SCFAs, causing metabolic disturbances involved in the pathology of PCOS. Decreased SCFA levels in patients with PCOS induce metabolic disturbances in the body by affecting the metabolism of glucose, mediating inflammatory processes, and thereby contributing to obesity. Dietary analyses of PCOS patients and healthy people revealed that dietary fiber and vitamin D intake were significantly lower in PCOS patients and that the number of gamma-aminobutyric acid (GABA)-producing bacteria was increased in PCOS patients and was positively correlated with the luteinizing hormone (LH)/follicular stimulating hormone (FSH) ratio. Gut microbes promote fat accumulation and thus induce obesity by inhibiting the expression of fasting-induced adipokines. Gut microbes influence the progression of MS and PCOS by affecting metabolic levels and regulating intestinal motility, glucolipid metabolism, and fat storage processes.

#### 4.2.5 Involvement of the gut microbiota in the progression of PCOS via the gut−brain axis

Disorders of the gut microbiota may be involved in the progression of PCOS via the gut−brain axis (Liang et al., 2021). The gut–brain axis regulates appetite, food intake, glucose metabolism, and energy maintenance (Li et al., 2023c) (Lach et al., 2018). Intestinal parasitic bacteria can produce SCFAs, which are involved in ghrelin secretion (Wang et al., 2023b). SCFAs regulate ghrelin expression by activating the mTOR pathway via G protein-coupled receptor (GPCR) 43 (Zhao et al., 2018). Ghrelin is involved in regulating LH secretion; ghrelin can inhibit the excessive synthesis and release of LH by delaying its release from the pituitary gland and is thus involved in regulating the function of the reproductive system in PCOS (Nohr et al., 2015). A study in which a letrozole-induced PCOS model was used to assess the effects of Lactobacillus plantarum on the brain−gut axis revealed that Lactobacillus plantarum CCFM1019 attenuated pathological changes in the ovary and restored testosterone and LH levels while altering the gut microbiota diversity and the relative abundance of bacteria that produce SCFAs. The rats in the CCFM1019 treatment group presented increased butyric acid levels while the extent of liver damage was reduced by altering the expression of GPCR41, which may be related to a butyric acid-dependent brain−gut axis mechanism (Cryan et al., 2019). Studies on the involvement of the gut microbiota in the progression of PCOS through the gut−brain axis are limited, and the specific mechanisms involved still require further investigation.

## 5 The gut microbiota and EMs

### 5.1 Overview of EM

EMs is a disease in which endometrial glands and stroma with normal growth function attach to and grow outside the uterine cavity, with progressive worsening dysmenorrhea, dyspareunia, chronic pelvic inflammatory disease, and infertility as the primary clinical symptoms (Horne and Saunders, 2019). EMs can be characterized by the following symptoms: dysmenorrhea, pain during sexual intercourse, chronic pelvic inflammation, and infertility (Bulletti et al., 2010). Although EMs are benign, they are malignant tumors characterized by adhesion, invasion, and metastasis. It is prone to recurrent attacks, which seriously affect the life and physical and mental health of female patients (Louis et al., 2011). The specific pathogenesis of EMs is still unclear, and there are mainly the endometrial implantation theory, retrograde flow of menstrual blood theory, epithelial chemotaxis theory, and genetic expression difference theory (Moradi et al., 2021). The doctrine of retrograde menstrual flow, proposed by Sampson, is thought to be the prevailing pathogenesis of EMs (Sampson, 1927). Up to 90% of women of childbearing age have menstrual blood reflux, but only 10% develop EMs, suggesting that other factors may be involved in the development of EMs (Becker et al., 2017). Multiple factors, such as inflammation, immunity, endocrinology, genetics, the environment, and metabolism, coordinate and promote each other, leading to the occurrence and development of EMs. Because of the comparative proximity of EMs to the intestinal tract and their malignancy-like invasive and recurrent properties, many studies have shown that EMs are closely linked to the functional homeostasis of the gut microbiota (Huang et al., 2021). Compared with normal controls, EMs patients have a greater Firmicutes/Bacteroidetes ratio (Le et al., 2021). Another study also revealed that, compared with those in the control group, the abundances of Actinobacteria, Firmicutes, Proteobacteria, and Verrucomicrobia in the gut microbiota of EMs patients were significantly greater, whereas the abundance of Lactobacillaceae was significantly lower (Leonardi et al., 2020). The significantly increased microorganisms in the gut microbiota of EMs patients are mainly gram-negative bacteria. Therefore, there is a close correlation between alterations in the gut microbiota and the onset of Ems (Shan et al., 2021).

### 5.2 Mechanisms of Gut Microbiota Involvement in EMs

#### 5.2.1 Involvement of the gut microbiota in immune system regulation in patients with EMs

The interaction between the immune system and the gut microbiota is fundamental for maintaining immune homeostasis (Zhang et al., 2022). EMs are characterized by autoimmune disorders such as reduced apoptosis, elevated cytokine levels, and cell-mediated abnormalities; thus, EMs are closely related to immune disorders (Kagbo-Kue et al., 2018). Immune imbalance is one of the most critical features of ectopic lesions and can lead to the development and exacerbation of symptoms of pain and infertility in patients (Vallve-Juanico et al., 2019). Toll-like receptor (TLR)4 is an essential receptor that is recognized by LPS and protects the host from bacterial and viral infections (Karen, 2015). The gut microbiota can cause EMs by influencing immune regulation. *E. coli* contamination of cyclic retrograde menstrual blood in EMs patients may be a persistent source of LPS in the peritoneal fluid (Khan et al., 2010). In turn, LPS produced by *Escherichia coli* may cause a proinflammatory response in the pelvis and the growth of endometriotic foci through the LPS/TLR4 cascade. Inflammatory factors produced by LPS can inhibit T-cell activation in the local inflammatory microenvironment through activation of the programmed cell death protein 1 (PD-1) and programmed cell death-ligand 1 (PD-L1) signaling pathways, leading to a reduction in the body’s immune capacity and allowing ectopic endometrial cells to escape immune cells (Wu et al., 2019). One study revealed that the growth of ectopic foci continued even in ovariectomized animals, suggesting that the innate immune system in the pelvic environment can also regulate the growth of ectopic foci in EMs. A deficiency of the immune system leading to difficulties in removing ectopic endothelial tissue is an essential factor in the pathogenesis of EMs.

#### 5.2.2 Dysregulation of the gut microbiota and the inflammatory microenvironment

The inflammatory response is a central process in the development of EMs. Increased proinflammatory cytokines in the peritoneal fluid of patients with EMs and pain-related stress increase intestinal permeability. In the event of intestinal barrier disruption, intestinal microbes infiltrate the intestinal epithelium and stimulate an immune response guided by intestinal mucosal dendritic cells (DCs), which induces defensive secretion of IgA, aggregation of neutrophils toward the site of inflammation, and an increase in the number of macrophages in the peritoneal cavity, resulting in the generation of an inflammatory microenvironment (Campbell et al., 2020). A decrease in the blood Treg/Th17 cell ratio was found after the induction of EMs, which may drive intestinal bacterial changes and thus promote disease progression by producing inflammatory mediators (Le et al., 2022). The inflammatory microenvironment is closely associated with the development of EMs, and the estrogen-driven inflammatory response is a central process in the formation of EMs, leading to pain, tissue remodeling, fibrosis, adhesion formation, and infertility. SCFAs can mediate the anti-inflammatory activity of macrophages and DCs, promote the differentiation and development of Treg cells, alleviate the inflammation of EMs through GPCRs, and inhibit histone deacetylase (HDAC) activity (Chadchan et al., 2021; Li et al., 2022b; Salliss et al., 2022).

#### 5.2.3 The gut microbiota is involved in the development of EMs by regulating circulating estrogen levels

Estrogen is a significant regulator of gut microbes, and the gut microbiota gene pool that metabolizes estrogen is known as the “estrogen metabolome” (Dabek et al., 2008). EMs are hormone-dependent diseases, and high levels of estrogen are directly associated with the development of Ems (Yuan et al., 2018). Gut microbes are involved in the estrogen cycle, forming the estrogen−gut microbe axis (Chen et al., 2017). Beta-glucuronidase and beta-glucosidase enzymes produced by Bacteroides, bifidobacteria, and lactobacilli in the intestinal tract promote estrogen catabolism and increase the reabsorption of free estrogen, leading to high circulating estrogen levels. The gut microbiota regulates estrogen levels through the production of SCFAs: butyric acid is one of the most abundant SCFAs, and butyric acid can regulate P and E2 synthesis in granulosa cells through the cAMP signaling pathway, which in turn promotes estrogen synthesis (Ata et al., 2019). The gut microbiota can synthesize estrogen-like compounds from dietary sources, which enhances the body’s estrogenic effects, thereby promoting the development of EMs. Increased estrogen levels can stimulate the growth and inflammatory activity of ectopic lesions. High estrogen exposure due to the gut microbiota may be a risk factor for the development of EMs. There were significant differences in the expression of 17β-E2 and 2-hydroxyestrone between patients with EMs and healthy individuals, and there was a significant positive correlation between the gut microbiota and urinary estrogen in patients with EMs. Studies in men and postmenopausal women have shown that the urinary levels of estrogen and most estrogen metabolites are closely related to the abundance and alpha diversity of the fecal microbiota, suggesting that the gut microbiota is closely related to estrogen metabolism *in vivo*. Research has shown that estrogen may play a role in regulating the microbial flora and immune metabolism in endometriosis (Alghetaa et al., 2023).

## 6 The gut microbiota and POF

### 6.1 Overview of POF

POF refers to ovarian failure in women younger than 40 years of age for various reasons and is the end stage of premature ovarian insufficiency (POI), which manifests as amenorrhea, infertility, FSH>40 IU/L and decreased estrogen levels (Di-Battista et al., 2020). Patients with POF often suffer from hot flashes, night sweats, osteoporosis, and other symptoms, and a lack of estrogen also causes metabolic disorders in the body; the risk of cardiovascular disease increases significantly, and a series of psychological problems, such as anxiety and depression, develop (Caserta et al., 2013). The increasing prevalence of POF in recent years, together with a trend toward rejuvenation, has had a severe impact on the quality of life of women and their physical health (Giudice and Kao, 2004). The pathogenic factors of POF are complex and diverse and include chromosomal abnormalities, the environment, oxidative stress, immune factors, metabolic disorders, and psychological factors (Schuh-Huerta et al., 2012; D’Avila et al., 2015). The pathogenesis of POF is closely related to the SIRT signaling pathway, TGF-β/Smad signaling pathway, PI3K/AKT signaling pathway, and Wnt/β-catenin signaling pathway (John et al., 2009; Tatone et al., 2018). Certain unhealthy habits, such as smoking, excessive alcohol consumption, and staying up late, can accelerate the process of POF. The specific etiology of POF has not been fully elucidated, and there is no satisfactory individualized treatment plan (Rahman and Panay, 2021). Clarifying the pathogenesis of POF and selecting the optimal treatment plan are the most important tasks for medical personnel. Ovarian endocrine levels can indirectly reflect ovarian function. The body regulates the secretion of various hormones through the hypothalamic−pituitary−gonadal axis. The hypothalamus acts on the pituitary gland through the secretion of gonadotropin release hormone (Gn RH), which releases FSH and stimulates the secretion of E2 through a positive feedback effect on the ovaries. When E2 is too high, the levels of Gn, RH and FSH are regulated through negative feedback to maintain the dynamic balance of hormones in the body. Estrogen levels in the blood are associated with the gut microbiota, and dysbiosis affects the enterohepatic circulation in mice, influencing the conversion of bound estrogen to free estrogen (Noguera-Julian et al., 2016). The gut microbiota and its metabolites are closely related to the development of autoimmune and metabolic diseases. Autoimmune abnormalities are essential factors in the pathogenesis of POF, and fluctuating sex hormone levels are among the critical clinical manifestations of POF (Jagarlamudi et al., 2010). Abnormal microbiota imbalances can affect immune cytokines and estrogens, ultimately leading to the development of POF (Fayed, 2015; Jeong et al., 2017). The gut microbiota participates in the regulation of sex hormones through direct or indirect pathways. It can also ameliorate POF by affecting the expression of immune-related cytokines such as Tregs, IFN-γ, and Th17 cells (Adlercreutz et al., 1984).

### 6.2 Gut microbiota-immune cytokine-POF

The gut microbiota promotes the synthesis of intestinal mucosal immunoglobulins, which regulate each other to maintain the homeostasis of the intestinal mucosal immune system. Relationships between the gut microbiota and immune cytokines, such as Tregs, IFN-γ, and Th17 cells, have been demonstrated (Fujimura et al., 2016; Han et al., 2019; Ji et al., 2019). On the basis of the role of immune cytokines in the gut microbiota and POF, immune cytokines are used as a bridge to connect POF and the gut microbiota to explore the relationship between POF and the gut microbiota: (1) There is a correlation between the gut microbiota, POF, and Treg cells. The gut microbiota promotes the expression and differentiation of Treg cells, mediates the participation of Treg cells in anti-inflammatory responses, and influences immune and metabolic homeostasis in the body. Clinically, POF patients exhibit changes in Treg numbers and improved immunomodulation after treatment (Russell, 1997). Human amniotic epithelial cells restore ovarian function by increasing the number of Treg cells in the spleens of AOD mice and regulating the function of activated macrophages in a paracrine manner to reduce inflammatory responses (Zhang et al., 2019b). Studies using human adipose-derived mesenchymal stem cells in combination with estrogen in POF patients have shown that human adipose-derived mesenchymal stem cells in combination with estrogen treatment have an immunomodulatory effect that promotes the proliferation of Tregs and improves impaired ovarian function (Song et al., 2018). (2) There is a correlation between the gut microbiota, POF and IFN-γ. Treatment with the gut microbiota can affect serum IFN-γ levels. IFN-γ can promote granulosa cell MHC class II antigen expression and stimulate an autoimmune response, leading to follicular atresia and POF (Coulam and Stern, 1991). A study in which human placental mesenchymal stem cells were transplanted into POF mice revealed that the decrease in serum TGF-β and increase in IFN-γ were reversed, suggesting that the restoration of ovarian function is related to the production of TGF-β and IFN-γ in POF mice (Yin et al., 2018). (3) There is a correlation between the gut microbiota, POF and Th17 cells. The gut microbiota affects immune function by regulating Th17 cells. The gut microbiota influences the body’s immune function by regulating Th17 cells; thus, the PI3K/Akt signaling pathway is involved in the restoration of ovarian function by altering the Th17/Tc17 and Th17/Treg cell ratios in POF mice after the transplantation of human mesenchymal stem cells.

### 6.3 The gut microbiota−HPO axis and estrogen−POF

The gut microbiota can affect ovarian function through the HPO axis. The hypothalamus secretes GnRH, which binds to pituitary GnRH-a and promotes pituitary secretion of LH and FSH, which act on the gonads to stimulate the synthesis and secretion of the steroid hormones testosterone, E2, and P. These hormones are also known as estrogens. Thus, the gut microbiota is closely related to ovarian function (Tetel et al., 2018). The gut microbiota affects ovarian function through estrogen levels. A decrease in Firmicutes and Bacteroidetes has been shown to increase serum glucagon-like peptide-1 (GLP-1) expression (Hwang et al., 2015). GLP-1 is an intestinal hormone and one of the stimulators of GnRH neurons and can influence GnRH secretion by modulating kissing peptide neurons (Outeirino-Iglesias et al., 2015). The GnRH pathway is a critical pathway involved in the regulation of reproductive function. The gut microbiota metabolites SCFAss and bile acids are both potent regulators of hypothalamic GnRH neuron function (Liao et al., 2021). Decreased gut microbiota diversity and an increased Firmicutes/Bacteroidetes ratio cause the gut microbiota to be ecologically imbalanced and secrete less glucuronidase activity. An imbalance in the gut microbiota leads to a decrease in estrogen and progesterone serum levels. A decrease in estrogen levels is one of the critical factors in the development of POF. Estrogen directly stimulates follicular development and can also indirectly affect ovarian function via negative feedback through the HPO axis, affecting the release of GnRH.

## 7 The gut microbiota provides potential treatments for infertility-related diseases

### 7.1 Gut microbiota and PCOS treatment

Probiotic therapy to restore gut microbiota homeostasis has been used with some success in treating female reproductive disorders (Karamali et al., 2018). Inulin and metformin can reduce the weight of mice, decrease the level of testosterone, and increase the level of E2 by altering the composition of the gut microbiota and inhibiting inflammation, altering the morphology of the ovaries (Huang et al., 2022). Probiotics restored the diversity of the gut microbiota in rats with PCOS, further improving the reproductive function of the rats (Cozzolino et al., 2020). Dietary improvements, as well as probiotic therapy, have been shown in clinical studies to positively impact the metabolic profile of women with PCOS, such as lower body weight and improved IR and lipid metabolism profiles (Jakubowicz et al., 2013). However, the types and doses of probiotics used in these studies vary widely, and further standardization is needed for future clinical studies. By studying the phenotype of prenatal androgenized mice, it was found that the appearance of gut microbiota abnormalities preceded the appearance of a PCOS-like phenotype in prenatal mice compared with controls, suggesting that the early gut microbiota is a potential target for the prevention of PCOS. Fecal microbiota transplantation (FMT) technology is gradually improving disease quality. Both fecal microbe transplantation and Lactobacillus transplantation in mice were found to decrease serum androgen levels, increase serum estrogen levels, improve ovarian dysfunction, and improve the estrous cycle (Yanjie et al., 2016). FMT has not been studied in the PCOS population. Further studies of FMT may provide novel alternative treatment options for PCOS.

### 7.2 Microecological agents against EMs

The gut microbiota structure and function are specific to patients with EMs, and treatments targeting the gut microbiota structure and metabolites, such as probiotics, antibiotics, and α-linolenic acid, have shown promising results (Ni et al., 2021). The difference in the levels of IL-1 and IL-6 produced by peripheral blood mononuclear cells in EMs patients and healthy controls was statistically significant, and the application of *Lactobacillus acidophilus* induced the production of IL-1 and IL-6; therefore, probiotics can be used to treat EMs patients. Probiotics can improve neurotransmitter synthesis and signaling in the gut microbiota, modulate neurotransmitter levels, affect pain pathways, and reduce pain perception in patients with Ems (Khodaverdi et al., 2019). Lactobacillus gasseri OLL2809 inhibits the development of ectopic endothelial cells by activating natural killer cells. The administration of Lactobacillus gasseri OLL2809 for three months significantly reduces dysmenorrhea (Itoh et al., 2011). In animal models, broad-spectrum antibiotic treatment has been shown to be effective in the treatment of EMs. Chadchan et al. reported that the use of antibiotics to remove Bacteroidetes inhibited the growth of ectopic endometrial foci in mice, suggesting that antibiotics may have the potential to prevent the progression of EMs by altering the gut microbiota to improve the inflammatory microenvironment. When mice with reduced ectopic foci were transplanted with fecal bacteria from endometriosis model mice, the ectopic foci of the former mice were significantly enlarged, suggesting that specific gut microbiota can promote the development of EMs (Chadchan et al., 2019). Exogenous supplementation with the bacterial metabolite unsaturated fatty acid α-linolenic acid improved the gut microbiota structure, dominant bacterial abundance, and intestinal wall barrier in EMs mice; regulated the intraperitoneal LPS content and inflammatory environment; and improved EMs. The use of gut microbiota preparations for diagnosing and treating EMs has broad research prospects (Pascale et al., 2019). No studies have reported the use of FMT for the treatment of EMs, and further exploration is needed. The combination of antibiotic treatment with other conventional therapies may be a potential treatment option for combating EMs.

### 7.3 The gut microbiota provides a new scientific basis for POF prevention and treatment

Studies on POF and the gut microbiota are relatively limited. The diversity of the gut microbiota was significantly greater in POF mice than in normal mice, with a low abundance of Helicobacter, Odoribacter, and Alistipes and a high abundance of Clostridium XIVa, Barnesiella, and Bacteroides (Cao et al., 2020). A comparison of the gut microbiota between POF patients and healthy women revealed that Firmicutes were more abundant in the intestines of healthy women. Moreover, Bacteroidetes, Butyricimonas, Dorea, and Lachnobacterium are more abundant in the intestines of POF patients (Wu et al., 2021). Dysregulation of the gut microbiota plays a vital role in the pathogenesis of POF (Wang et al., 2020). During cyclophosphamide-induced POF, the mouse gut microbiota is significantly altered, with a decrease in Akkermansia abundance and a marked increase in Lactobacillus abundance (Lin et al., 2020). Fisetin attenuates cyclophosphamide-induced ovarian damage by modulating the gut microbiota in a manner that decreases CCR9+, CXCR3+, CD4+, T lymphocytes, and IL-12. The factors contributing to POF do not exist independently but interact with each other and synergistically contribute to the accelerated progression of ovarian senescence. The gut microbiota affects the occurrence and development of POF through various pathways and factors, and the underlying mechanism needs to be further explored. In the future, further exploration should be conducted to identify the characteristics of the gut microbiota profile in patients with POF, as well as to discover specific microbial spectra associated with the onset and progression of POF. These findings provide a deeper understanding of the pathogenesis of POF from metabolic, inflammatory, and other perspectives, ultimately leading to the development of effective treatment strategies.

## 8 Conclusion

Infertility is a public health problem that has a significant effect on women’s quality of life and reproductive health, as well as on economic and social development and population security. The gut microbiota can affect the development of infertility in various ways. Dysregulation of the gut microbiota leads to an increase in intestinal permeability, resulting in an increase in lipopolysaccharide levels in the body, triggering inflammatory and immune responses in the body, resulting in disruption of glucose metabolism in the body and disruption of the gut microbiota, which ultimately leads to the development of infertility symptoms in the patient (**Table 1**). Intervening in infertility by regulating the gut microbiota through probiotics, nutrients, antibiotics, and FTM supplementation provides new ideas for treating infertility. The many roles of the gut microbiota in the pathogenesis of infertility disorders are well documented, but certain limitations remain: (1) studies on the relationship between the gut microbiota and infertility are focused primarily on the correlation level, and there are still few studies on its specific mechanism of action; (2) the influence of regional, dietary, ethnic, and cultural differences on the structural composition of the gut microbiota is a significant interfering factor in related studies; and (3) there are individual differences in the number and types of human gut microbiota, and individualized application is an important issue that needs to be explored in further research in the future. Future exploration of the potential mechanisms by which gut microbiota alterations mediate infertility is needed to provide new strategies for the prevention, diagnosis, and treatment of infertility.

**Table 1:** Changes in the gut microbiota associated with infertility-related diseases.

Disease	Sample source	Main findings	References
PCOS	human	the beta diversity of microbiomes in women with PCOS was significantly decreased, and *B. vulgatus* was markedly increased	(Qi et al., 2021)
PCOS	mice	PCOS mice exhibited reduced abundances of gut microbiome species, as well as decreased phylogenetic diversity, with significantly higher levels of Firmicutes compared to control mice	(Qi et al., 2019)
PCOS	human	The abundance of the *Tenericutes* phylum in women with PCOS was significantly lower	(Kelley et al., 2016)
PCOS	human	In PCOS women, there was a decrease in Akkermansia and Ruminococcaceae, while gram-negative bacteria belonging to the Bacteroides and Escherichia/Shigella genera were significantly increased	(Zhou et al., 2020)
PCOS	human	The gut microbiome of PCOS women experienced a significant increase in gram-negative bacteria belonging to the genera Bacteroides and Escherichia/Shigella	(Liu et al., 2017)
PCOS	human	Bilophila, Blautia, and Holdemania exhibited a protective effect against PCOS, whereas the Lachnospiraceae family of bacteria was associated with detrimental effects in individuals with PCOS	(Li et al., 2023a)
PCOS	human	Women with PCOS have reduced alpha diversity in the gut microbiota compared to healthy people, and the relative abundance of Bacteroidaceae was significantly increased	(Zou et al., 2023)
PCOS	human	women with PCOS possess significantly lower microbial alpha diversity compared with controls	(Sola-Leyva et al., 2023)
PCOS	human	The depletion of *Lachnospira* and *Prevotella* and enrichment of *Bacteroides*, *Parabacteroides*, *Lactobacillus*, *Fusobacterium*, and *Escherichia/Shigella* in PCOS	(Li et al., 2023b)
EMs	mice	*Firmicutes*/*Bacteroidetes* ratio was elevated, and *Bifidobacterium* was also increased	(Jess et al., 2012)
EMs	mice	the Firmicutes/Bacteroidetes ratio was elevated in mice with endometriosis	(Yuan et al., 2018)
EMs	human	More women in the stage 3/4 endometriosis group had *Shigella/Escherichia* dominant stool microbiome	(Ata et al., 2019)
EMs	mice	mice with endometriosis had more Bacteroidetes and less Firmicutes in their guts than mice without endometriosis	(Chadchan et al., 2019)
EMs	human	the EM group had a lower α diversity of gut microbiota and a higher *Firmicutes*/*Bacteroidetes* ratio	(Shan et al., 2021)
EMs	human	There were differences in abundance of 12 genus belonging to the classes Bacilli, Bacteroidia, Clostridia, Coriobacteriia, and Gammaproteobacter between endometriosis patients and controls	(Svensson et al., 2021)
EMs	mice	The increased abundance of chenodeoxycholic and ursodeoxycholic acids and the decreased abundance of ALA and 12,13-EOTrE were found in the feces of EMS mice	(Ni et al., 2020)
POF	mice	the proportions of Helicobacter, Odoribacter, and Alistipes were lower in the dominant flora of the POF group, while Clostridium XIVa, Barnesiella, Bacteroides, and Mucispirillum were higher	(Cao et al., 2020)
POF	mice	During CTX induced POF, the abundance of Akkermansia decreased while the abundance of Lactobacillus increased	(Lin et al., 2020)
POI	human	higher *Butyricimonas*, *Dorea*, *Lachnobacterium*, and *Sutterella* and lower *Bulleidia* and *Faecalibacterium* abundances at the genus level were observed in women with POI	(Jiang et al., 2021)
POI	human	*E. hallii* and *E. ventriosum* have protective effects against POI, whereas *Intestinibacter* and *Terrisporobacter* have detrimental effects on POI	(Wang et al., 2023a)
POI	human	phylum Bacteroidetes, genera Butyricimonas, Dorea, Lachnobacterium and Sutterella enriched significantly in women with POI	(Wu et al., 2021)

## Glossary

PCOS: polycystic ovary syndrome
EMs: endometriosis
POF: premature ovarian failure
SCFAs: short-chain fatty acids
LPS: lipopolysaccharide
HA: hyperandrogenism
IR: insulin resistance
MS: metabolic syndrome
GABA: gamma-aminobutyric acid
LH: luteinizing hormone
FSH: follicle stimulating hormone
GPCR: G protein-coupled receptor
FMT: fecal microbiota transplantation
TLR: Toll-like receptor
PD-1: programmed cell death protein 1
PD-L1: programmed cell death-ligand 1
DCs: dendritic cells
HDAC: histone deacetylase
GnRH: gonadotropin release hormone
GLP-1: glucagon-like peptide-1
